# Tunable Electric Properties of Bilayer α-GeTe with Different Interlayer Distances and External Electric Fields

**DOI:** 10.1186/s11671-018-2813-x

**Published:** 2018-12-07

**Authors:** Dingbo Zhang, Zhongpo Zhou, Haiying Wang, Zongxian Yang, Chang Liu

**Affiliations:** 1 Henan Key Laboratory of Photovoltaic Materials, and School of Physics and Materials Science, Henan Normal University, Xinxiang, 453007 China; 2 Key Laboratory of Artificial Micro- and Nano-structures of Ministry of Education, and School of Physics and Technology, Wuhan University, Wuhan, 430072 China

**Keywords:** α-GeTe, Bilayer, First-principle calculations, Electric properties

## Abstract

Based on first-principle calculations, the stability, electronic structure, optical absorption, and modulated electronic properties by different interlayer distances or by external electric fields of bilayer α-GeTe are systemically investigated. Results show that van der Waals (vdW) bilayer α-GeTe has an indirect band structure with the gap value of 0.610 eV, and α-GeTe has attractively efficient light harvesting. Interestingly, along with the decrease of interlayer distances, the band gap of bilayer α-GeTe decreases linearly, due to the enhancement of interlayer vdW interaction. In addition, band gap transition is originated from the electric field-induced near free-electron gas (NFEG) under the application of positive electrical fields. However, when the negative electric fields are applied, there is no NFEG. On account of these characteristics of bilayer α-GeTe, a possible data storage device has been designed. These results indicate that bilayer α-GeTe has a potential to work in new electronic and optoelectronic devices.

## Introduction

The success of graphene [1, 2] has stimulated tremendous research in novel two-dimensional (2D) materials, including hexagonal boron nitride (h-BN) [3], transition-metal dichalcogenides (TMDs) [4], transition-metal carbides (MXenes) and nitrides [5], and van der Waals (vdW) heterostructures [6]. These 2D materials can work in electronic or optoelectronic applications [7, 8] due to tunable electronic properties [9] and superior flexibility under tensile strain [10]. However, there are more or less challenges in 2D materials, such as the facile degradation of phosphorene in air [11], low hole mobility, and weak absorption of the visible light of indium selenide (InSe) [12], as well as the zero band gap of graphene [7], silicene [13], and germanene [14]. Therefore, it is necessary to investigate new 2D materials with outstanding stability, high carrier mobility, and desired band gap.

In the past years, bulk α-GeTe has been applied in various fields, such as nonvolatile phase-change memory technologies [15, 16], neuromimetic computing applications, and thermoelectrics [17, 18]. Recently, nanostructured α-GeTe has been widely fabricated by atomic layer deposition (ALD) [17], vapor–solid–liquid (VLS) methods [18], and chemical methods using surface-stabilizing polymers [19]. Nanostructured α-GeTe [20] phases have a higher crystallization temperature and a lower melting point than bulk α-GeTe [19]. Most importantly, α-GeTe is an IV–VI semiconductor with buckled atomic layers in which Ge and Te atoms are bonded. There is weak vdW force between the layers of α-GeTe.

Very recently, few-layer α-GeTe nanosheets of two to four layers and even monolayer α-GeTe were obtained through the application of sonication-assisted liquid-phase exfoliation to α-GeTe powder dispersed in ethanol by Zhang et al. [21]. However, few theoretical studies focus on the modulating electronic properties of 2D α-GeTe using external electric fields and vertical strain, both of which are the effective methods in band gap engineering [22]. Considering the fact, the multilayer structure is more available than monolayer in potential applications. So, the study of bilayer α-GeTe, which is the most typical multilayer structure, is essential to potential development of 2D α-GeTe nanosheet. In this paper, based on first-principle calculations, the stability, band structures, optical absorption, and modulated electronic properties by different interlayer distances and by external electric fields of bilayer α-GeTe are systemically investigated. Our studies prove that the vdW bilayer α-GeTe is potential for new electronic and optoelectronic devices.

## Computational Methods

All calculations are performed based on the spin-polarized density functional theory (DFT) using the projected-augmented wave (PAW) method implemented in Vienna Ab initio Simulation Package (VASP) [23, 24]. The generalized gradient approximation of Perdew-Burke-Ernzerhof (GGA-PBE) [25] is selected to describe the electron exchange and correlation. The vdW interaction is considered by using a semi-empirical DFT-D3 method [26]. The cutoff energy of plane wave is set to be 500 eV to ensure the convergence of total energy, and 15 × 15 × 1 k-point meshes are selected for Brillouin zone integration. To separate the interactions between the periodic slabs, the vacuum space in the *z* direction is set to 30 Å. The lattice vectors and atomic positions are fully relaxed until the force and energy are converged to 0.01 eV/Å and 10^−5^ eV, respectively. As GGA-PBE method usually underestimates the band gap of semiconductors, Heyd–Scuseria–Ernzerhof (HSE06) [27] method is employed to correctly calculate gap values and band edges for semiconductors. Thus, the electronic structures and optical properties are calculated by using the HSE06. The phonon band structure is performed by using the density functional perturbation theory (DFPT) as implemented in Phonopy [28], which adopts the quasi-harmonic approximation method to analyze the potential energy hypersurface in the neighborhoods of the minimum-energy structure.

## Results and Discussion

### Geometric Structure

**Fig. 1:**
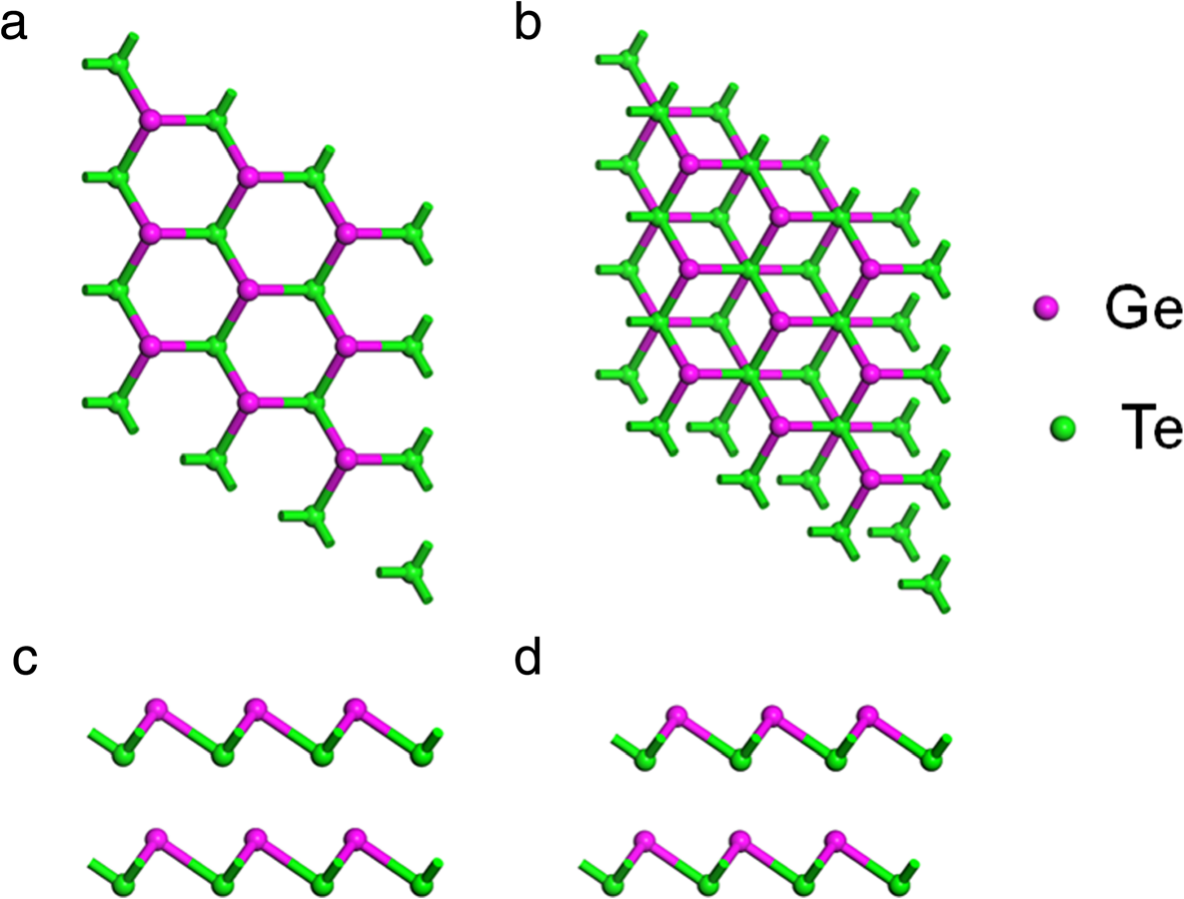
Top view (**a**) and side view (**c**) of AA-stacking bilayer α-GeTe. Top view (**b**) and side view (**d**) of AB-stacking bilayer α-GeTe

**Fig. 2:**
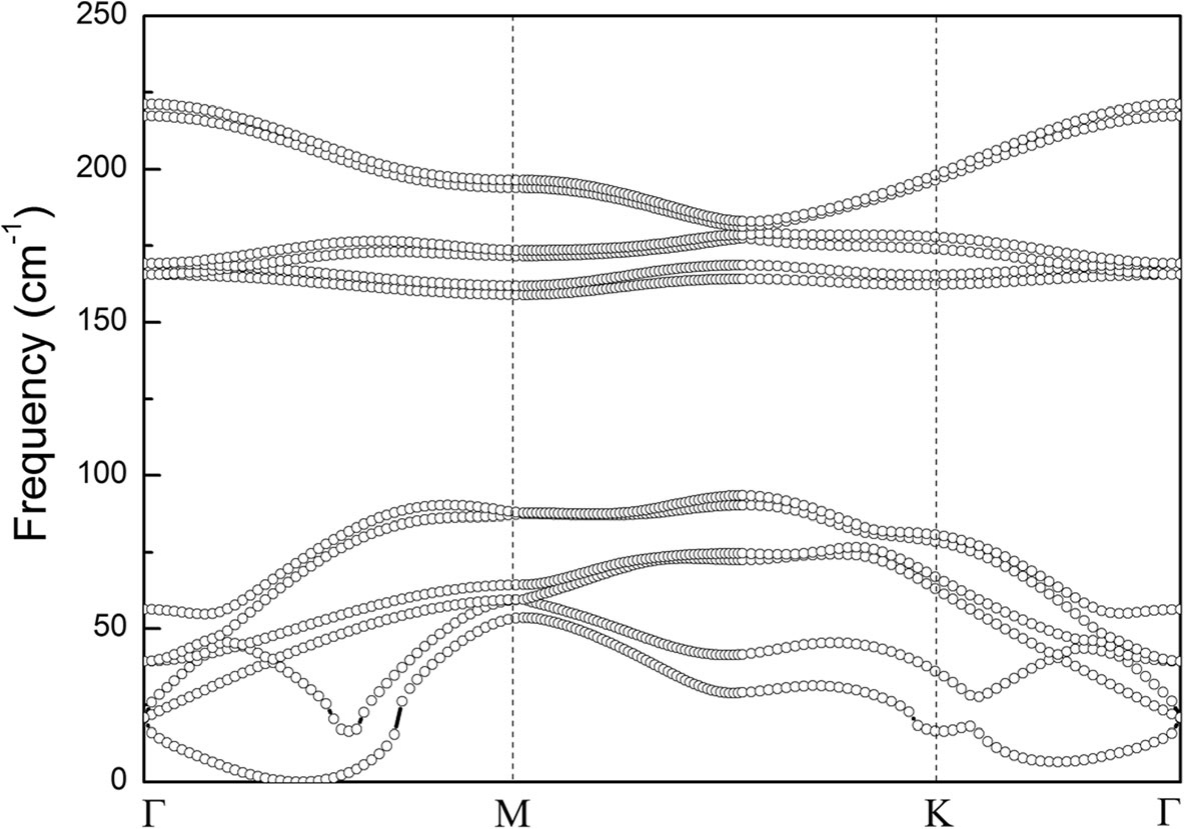
Phonon band dispersion of AA-stacking bilayer α-GeTe

Monolayer α-GeTe has the hexagonal structure with buckled atomic layers in which Ge atoms are located at one layer and Te atoms lie in the other layer. The optimized lattice parameters, bond lengths, and angles of monolayer α-GeTe are a = b = 3.95 Å, *L*_Ge-Te_ = 2.776 Å, and *θ* = 91.497°, respectively. The lattice parameter monolayer α-GeTe also agrees with a previous report [21]. For bilayer α-GeTe vdW heterostructures, two types of possibly high-symmetry stacking structures, namely AA- and AB-stacking, are considered, as shown in Fig. 1. AA-stacking exhibits a hexagonal stacking arrangement. AB-stacking has the Bernal stacking feature as the structure of bulk α-GeTe. The total energies of the two stacking structures are calculated to evaluate relative stability, respectively. The result shows that the total energy of AA-stacking is 147 meV less than that of AB-stacking. The more stable structure of the bilayer α-GeTe is AA-stacking, different from that of its bulk. Also, the calculated equilibrium distance is 2.920 Å for AA-stacking bilayer α-GeTe. The calculated phonon dispersion of AA-stacking bilayer α-GeTe, demonstrated in Fig. 2, indicates that AA-stacking bilayer α-GeTe is stable, due to no imaginary frequency in the phonon spectrum. In addition, the stable two-layer α-GeTe has been obtained in the experiment [21]. Thus, AA-stacking bilayer α-GeTe is mainly discussed in the following section.

### Electronic Structures

**Fig. 3:**
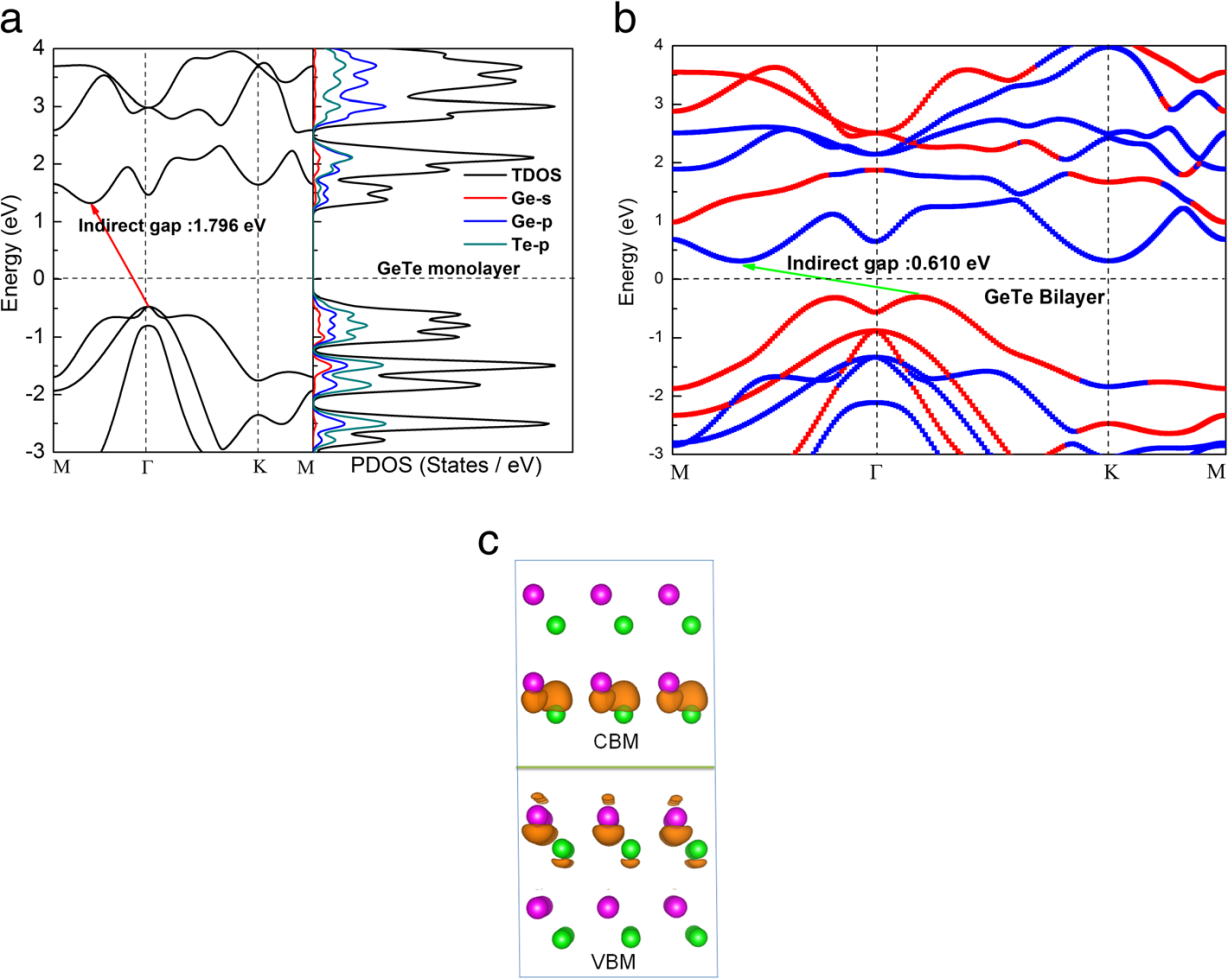
**a** Band structure and partial density of states of monolayer α-GeTe. Projected band structure (**b**) denoted by blue lines (down layer) and red lines (up layer) of bilayer α-GeTe. Band-decomposed charge density (**c**) of the VBM and CBM for bilayer α-GeTe

To understand clearly the electronic properties of bilayer α-GeTe, band structure and projected density of states (PDOS) of monolayer α-GeTe are calculated, as depicted in Fig. 3a. The conduction band minimum (CBM) lies between M and Γ points, while the valence band maximum (VBM) is located at Γ point, which indicates that monolayer α-GeTe is an indirect band gap semiconductor with the energy-gap value of 1.796 eV, in good agreement with previous results [21]. According to the PDOS, the CBM is largely composed of the states Ge-s, Ge-p, and Te-p, while the states in the VBM are attributed to the Ge-p and Te-p states. For the bilayer α-GeTe, the projected band structure is plotted in Fig. 3b, indicating an indirect band with the gap value of 0.610 eV. The CBM of bilayer α-GeTe is dominated by down layer, lying between M and Γ points, while the VBM is mainly contributed by the states from the up layer, being located at between Γ and K points. There is an interesting thing that projected band structure of bilayer α-GeTe seems to be the sum of the monolayer component, which indicates that a typical weak vdW interaction exists in bilayer α-GeTe. To gain further insight into bilayer α-GeTe, the band-decomposed charge density of the VBM and CBM are calculated, as shown in Fig. 3c. The band-decomposed charge density of the CBM and VBM are distinctly different. The states of lowest-energy electrons and the highest-energy holes are localized in the down layer and up layer, respectively, which cause the effective separation of electrons and holes with type-II band edge alignments. Therefore, the spatially indirect exciton recombination occurs through the staggered gap of bilayer, which is important for optoelectronic applications [12].

### Optical Properties

**Fig. 4:**
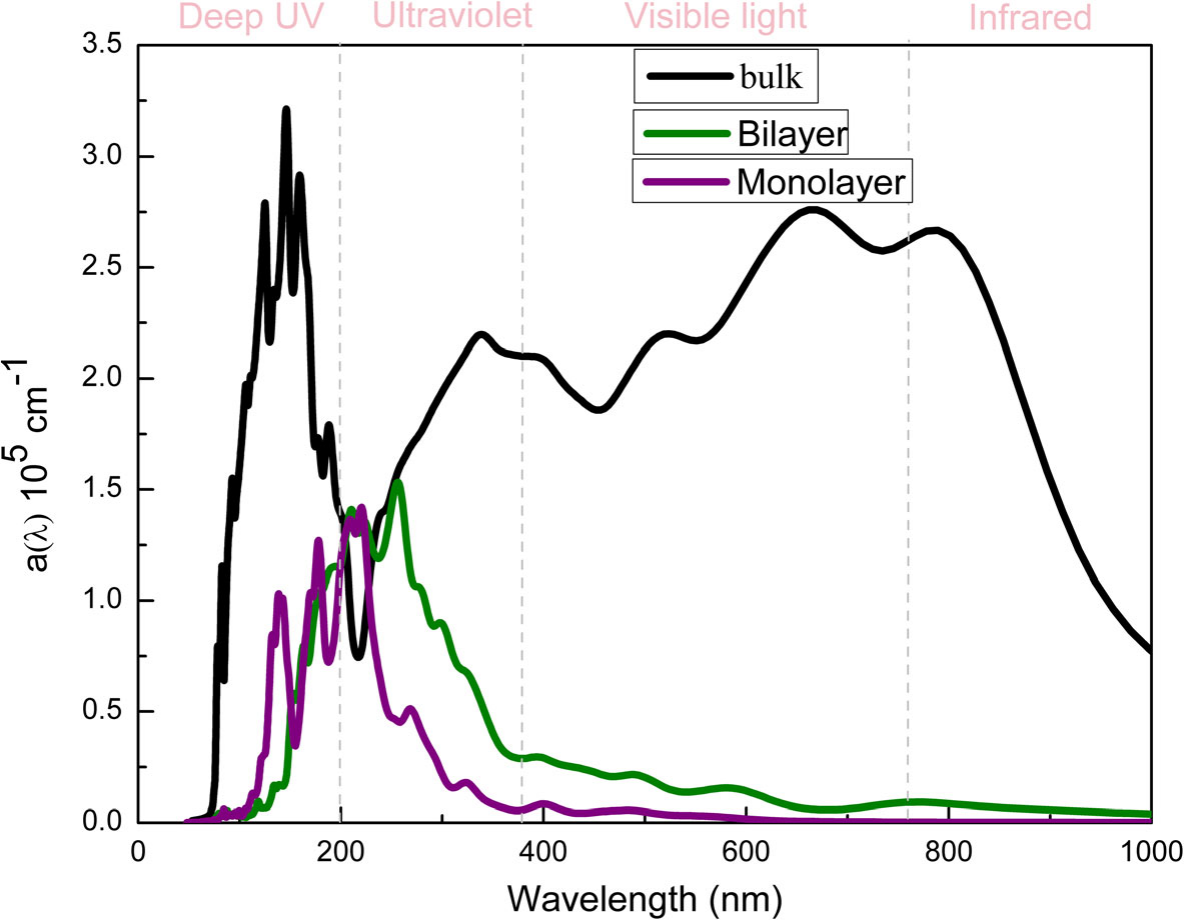
Absorption coefficient of monolayer and bilayer α-GeTe

It is very important to study optical absorption in optoelectronic devices. Based on the frequency-dependent dielectric function *ε*(*ω*), the optical absorption coefficient *a*(*ω*) of monolayer and bilayer α-GeTe can be calculated according to the formula [12, 22]:

$$ \alpha \left(\omega \right)=\sqrt{2}\omega {\left[\sqrt{\omega_1^2\left(\omega \right)+{\omega}_2^2\left(\omega \right)}-{\omega}_1\left(\omega \right)\right]}^{\raisebox{1ex}{$1$}\!\left/ \!\raisebox{-1ex}{$2$}\right.} $$

where *ε*_1_ (*ω*) and *ε*_2_ (*ω*) are the real part and imaginary part of the complex dielectric function, respectively. In Fig. 4, the obtained optical absorption coefficients of monolayer, bilayer, and bulk α-GeTe are demonstrated. Monolayer α-GeTe has three absorption peaks, in accordance with its transitions between the conduction band and the valence band of monolayer α-GeTe. And there is obvious light absorption in the ultraviolet and deep ultraviolet regions. However, bilayer α-GeTe has a distinct light absorption in the visible and infrared regions as well. Similar to bilayer α-GeTe, bulk α-GeTe exhibits broad optical absorption ranging from the deep ultraviolet to the infrared, and the optical absorption intensity can reach the order of 10^5^ cm^−1^. This enhanced optical absorption intensity is caused by the increased layer-number of bulk α-GeTe, comparing with monolayer and bilayer α-GeTe. Thus, α-GeTe might be promising materials for optoelectronics application due to the efficiency of solar energy utilization.

### Effect of Vertical Strain

**Fig. 5:**
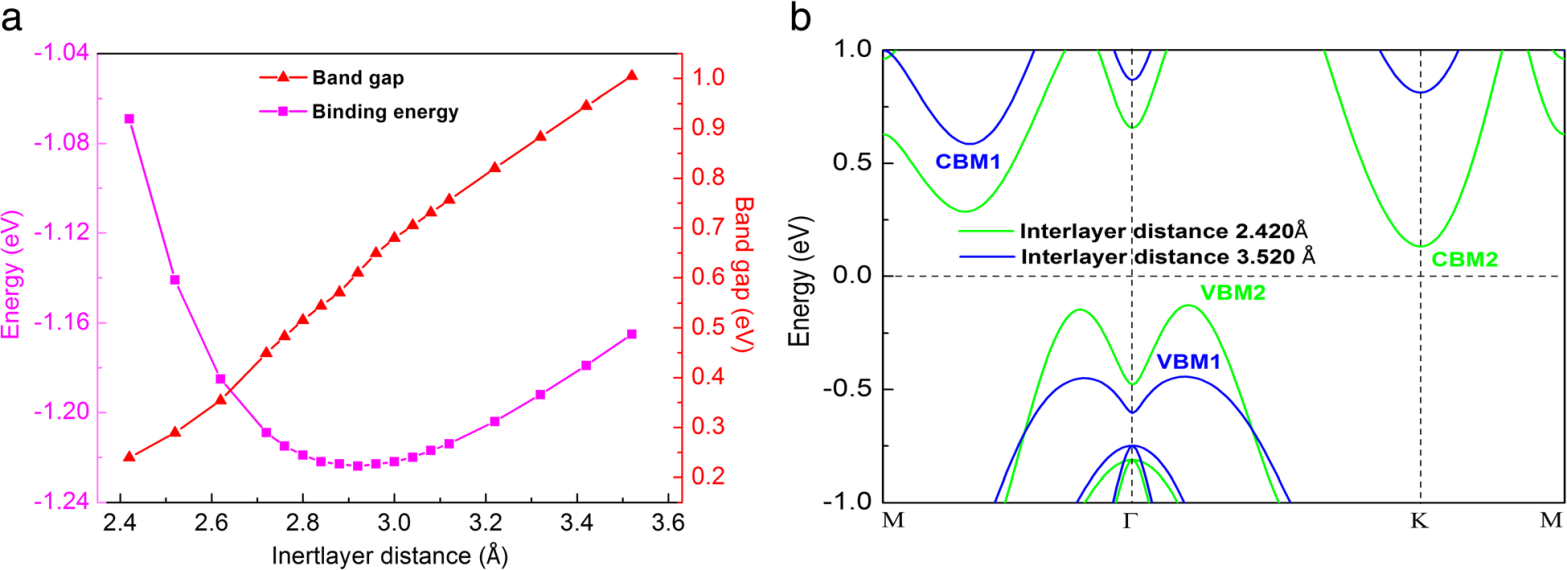
Variations of binding energy and band gap (**a**) of bilayer α-GeTe as function of interlayer distance. Band structures (**b**) of bilayer α-GeTe with 2.420 Å and 3.520 Å interlayer distances

Applying vertical strain is an effective way to modulate the electronic properties of bilayer materials. Figure 5a shows the band gap as function of the interlayer distances. The binding energy (*E*_b_) is calculated by the equation [22]:

$$ {E}_{\mathrm{b}}={E}_{\mathrm{b}\mathrm{ilayer}}-2{E}_{\mathrm{monolayer}} $$

where *E*_bilayer_ and *E*_monolayer_ are the total energies of bilayer and monolayer α-GeTe, respectively. With the interlayer distance varieties from 2.420 to 3.520 Å, the binding energies are all negative. More importantly, the distance with *d* = 2.920 Å corresponds to the minimum value of the *E*_b_, indicating the most stable structure. Moreover, the band gap of bilayer α-GeTe can be continuously tuned by the different interlayer coupling. Band gaps monotonically increase, but the shape of all band structures are kept unchanged with the distances varying from 2.420 to 3.520 Å. In Fig. 5b, band structures are plotted for bilayer α-GeTe with 2.420 Å and 3.520 Å interlayer distances. The CBM1 and VBM1 are corresponding with the interlayer distance 3.520 Å, and the CBM2 and VBM2 are related to the interlayer distance 2.420 Å. The CBM declines while the VBM rises along with the decreasing interlayer distances. The band gap increases with an increase in the interlayer distance for bilayer α-GeTe due to the enhancement of the vdW interlayer interaction and orbital overlapping. Similar behaviors can be found in bilayer InSe [22].

### Effect of External Electric Fields

**Fig. 6:**
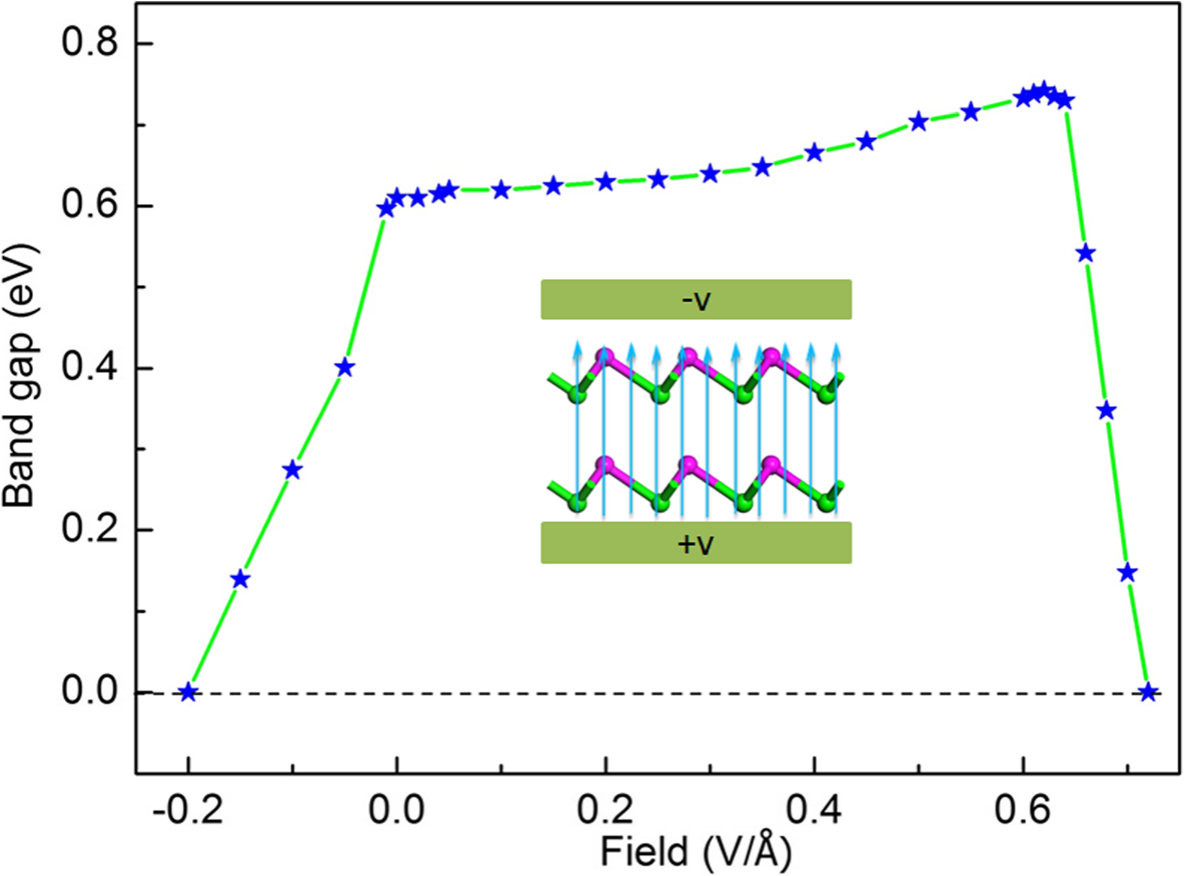
Variations of band gap of bilayer α-GeTe as a function of the applying vertical electric field. The colored horizontal dashed lines are shifted zero-gap

Another effective way to tune the electronic properties of 2D vdW bilayer is applying vertical external electric fields. In order to get valid results, a planar dipole layer is carried out in the middle of the vacuum region and the symmetry is called off in all calculations with the application of electric fields [29]. Moreover, the positive direction is defined as pointing from the down layer to the up layer. In Fig. 6, band gap of bilayer α-GeTe changes subtly, when the values of applying electric fields (*E*_app_) are varying from 0.01 to 0.64 V/Å. When *E*_app_ is less (or larger) than a critical value (*E*_c_), the band gap of bilayer α-GeTe drops very quickly and linearly. Then, the semiconductor-to-metal transition of bilayer α-GeTe occurs until *E*_app_ is less (or greater) than a typical value (*E*_t_). These results show that the larger the applied electric field strength, the stronger the hybridization between the two layers.

**Fig. 7:**
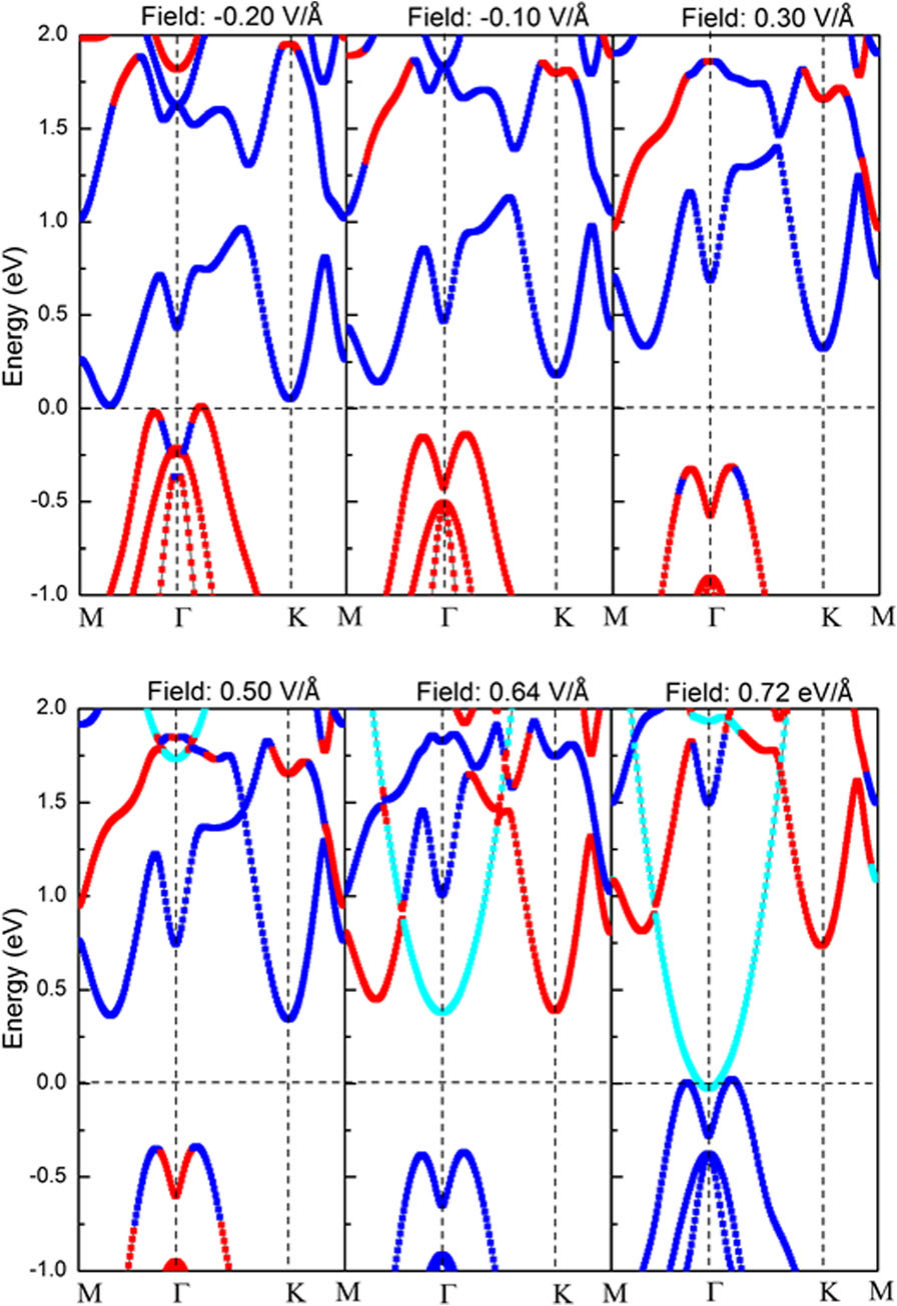
Projected band structure of bilayer α-GeTe denoted by blue lines (down layer) and red lines (up layer) under different external vertical electric fields

Notably, the range of *E*_c_–*E*_t_ is 0.01–0.20 V/Å with the application of negative electrical fields, which is distinctly larger than the range of *E*_c_–*E*_t_ (0.64–0.72 V/Å) with the positive applied electrical fields. To understand the band gap transition under the applied vertical electrical fields, projected band structures of bilayer α-GeTe under the selected external vertical electrical fields have been calculated, as shown in Fig. 7. When *E*_app_ = − 0.20 and *E*_app_ = − 0.10 V/Å, the CBM and the VBM of bilayer α-GeTe are also contributed by the down layer and up layer, respectively. The application of vertical electrical fields makes both the CBM and the VBM closer to the Fermi level, eventually achieving a semiconductor-metal transition at *E*_app_ = − 0.20 V/Å. On the other hand, with an increase in the positive applied electrical fields, the energy level of band structures of the down layer is gradually increased, and vice versa is observed for the up layer. As a result, the up layer and down layer are attributed to the CBM and the VBM of bilayer α-GeTe, respectively, when *E*_app_ ≥ 0.64 V/Å. Moreover, additional conduction bands appear under the applied positive electrical fields, which are indicated by the cyan line. These bands are not contributed by the down layer or up layer, which shows the near free-electron gas (NFEG) feature [30]. The energy level of the NFEG band falls very quickly with an increase of applied electrical field. When *E*_app_ ≥ *E*_c_ ~ 0.64 V/Å, the CBM consisted of the NFEG band. When *E*_app_ ≥ *E*_t_ ~ 0.72 V/Å, the NFEG band is close to the Fermi level, and the VBM of the down layer contact with the NFEG band, indicating the metallic band structure feature. And band gap variation tendency of bilayer α-GeTe under the positive application of electrical fields is analyzed further. For *E*_app_ < *E*_c_, the band gap depends on the energy level difference between the CBM and VBM, which is not sensitive to the application of electrical fields. Hence, band gap is relatively stable. For *E*_c_ < *E*_app_ < *E*_t_, the NFEG band takes over the CBM and dominates the band gap change. Band gap decreases sharply and linearly, as energy level of the NFEG band sharply drops. For *E*_app_ < *E*_t_, energy level of the NFEG band goes lower than that of the VBM. Hence, the semiconductor-metal transition of bilayer α-GeTe comes from the electric field-induced NFEG. Moreover, bilayer α-GeTe has more than twice the *E*_t_ of bilayer InSe [29], indicating that the semiconductor-metal transition of bilayer α-GeTe needs more voltage.

**Fig. 8:**
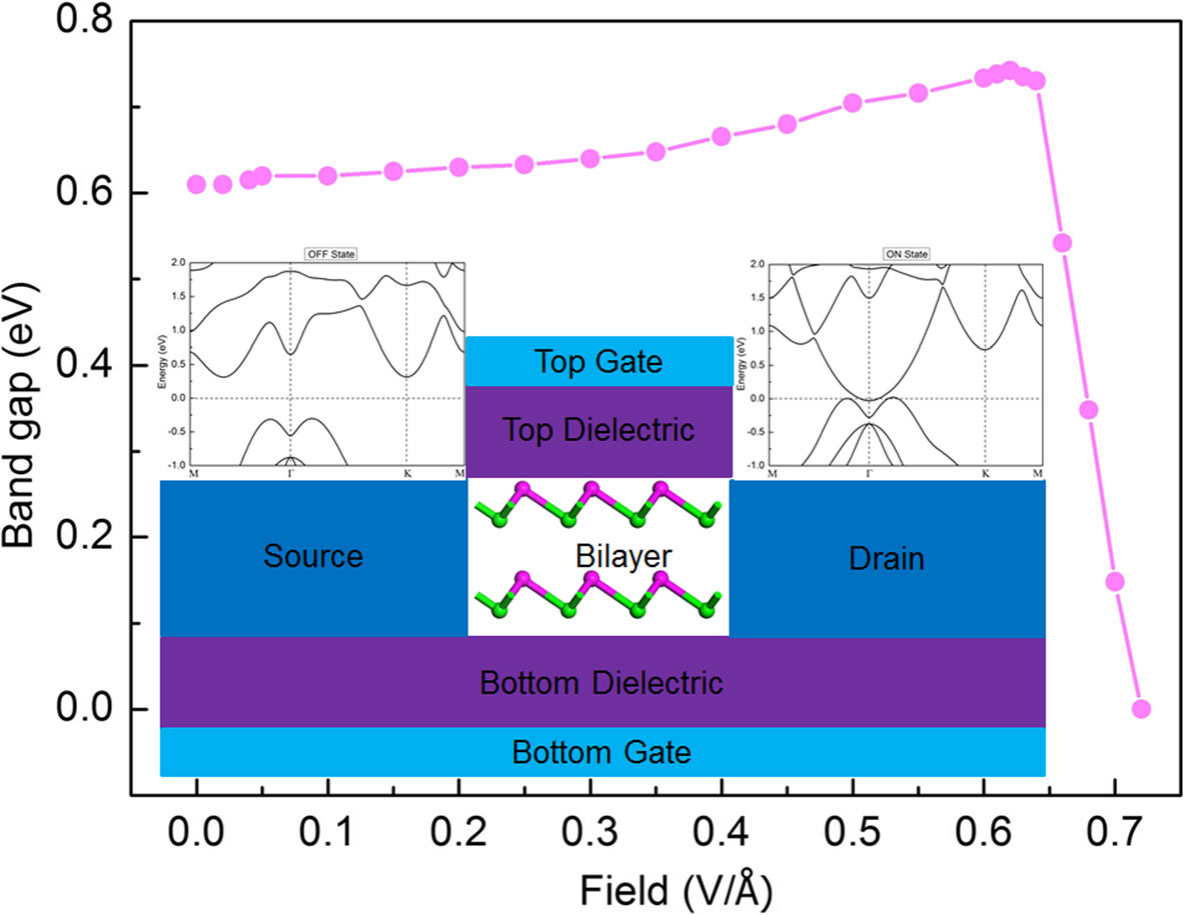
Band gap of bilayer α-GeTe as function of the applying electric field. Inset is the schematic model

A possible data storage device using bilayer α-GeTe has been designed based on the results above, whose schematic structure is built, as illustrated in Fig. 8. Bilayer α-GeTe is transferred to the thin Si/SiO_2_ substrates. The same Si/SiO_2_ layer is covered on the bilayer α-GeTe to protect 2D α-GeTe from the air. The large-area graphene film is transferred and used for the source and drain electrodes owing to its high optical transmittance and conductivity [31]. Native bilayer α-GeTe is a semiconducting with a high electrical resistance OFF state between the source and drain electrodes. The electric field-induced NFEG can modulate bilayer α-GeTe to be the zero gap by *E*_app_ ≥ *E*_t_ from the bottom to top Si, which implies zero electrical resistance ON state between the source and drain electrodes. The NFEG as well as the ON state can be kept within this field effect transistor (FET) device when the applying electric field is withdrawn. When the negative electrical field is applied, the NFEG in bilayer α-GeTe is erased. Therefore, the OFF and ON states with the semiconducting and metallic band structure features can be stored in bilayer α-GeTe-based data storage devices.

## Conclusion

In summary, the stability of bilayer α-GeTe is investigated by calculating the binding energies and phonon band dispersion based on vdW-corrected first-principle. The vdW bilayer α-GeTe has an indirect band gap with a typical type-II band alignment. Especially, α-GeTe has enhanced optical absorption range and intensity. Further, the band gap of bilayer α-GeTe can be tuned by the applying vertical strain and the applying external vertical fields. Only when the positive electric fields are applied, the NFEG exists. And the electric field-induced NFEG can make the band gap vary extremely quickly. Based on these outstanding characteristics, a possible data storage device based on bilayer α-GeTe is proposed. These results explain the underlying mechanism of band gap transition for bilayer α-GeTe. In all, the effective charge separation, broad optical absorption spectrum, high optical absorption intensity, and the NFEG feature make the bilayer α-GeTe potential material work in 2D material-based electronic and optoelectronic devices.

## Abbreviations

2D: Two-dimensional
ALD: Atomic layer deposition
CBM: Conduction band minimum
DFT: Density functional theory
*E*_app_: Values of applied electric fields
FET: Field effect transistor
GGA-PBE: Generalized gradient approximation of Perdew-Burke-Ernzerhof
h-BN: Hexagonal boron nitride
HSE06: Heyd–Scuseria–Ernzerhof
InSe: Indium selenide
MXenes: Transition-metal carbides
NFEG: Near free-electron gas
PAW: Projected-augmented wave
PDOS: Projected density of states
TMDs: Transition-metal dichalcogenides
VASP: Vienna Ab initio Simulation Package
VBM: Valence band maximum
vdW: van der Waals
VLS: Vapor–solid–liquid
